# Large cameral coronary artery fistula in a 5 months old infant with unusual presentation and fatal outcome: -case report

**DOI:** 10.1186/s12887-023-04196-7

**Published:** 2023-08-05

**Authors:** Tamirat Moges, Hayat Ahmed, Azmeraw Gisila

**Affiliations:** 1 Department of Pediatrics and Child Health, School of Medicine, College of Health Sciences Addis Ababa University, Addis Ababa, Ethiopia; 2 Department of Radiology School of Medicine, College of Health Sciences Addis Ababa University, Addis Ababa, Ethiopia

**Keywords:** Cameral coronary artery fistula, Congenital heart disease, Congestive heart failure, Cyanosis

## Abstract

**Background:**

Congenital coronary fistulas (CAFs) are uncommon abnormalities communicating the coronary arteries with the cardiac chambers or portion of the systemic or pulmonary circulation. Over 90% of the cases drain into the right side of the heart with only 3% terminating in the left ventricle. Infants with a large CAFs may develop congestive heart failure.

**Case presentation:**

A 5 months old female infant presented with labored breathing and worsening of bluish discoloration of the lips and extremities following a prolonged cry. She had a history of breastfeeding difficulty and noticeable bluish discoloration of the lips and extremities since birth. The infant was wasted and had a fast heart rate, bluish lips, and nail beds with clubbing of fingers and toes. A cardiac murmur was noted during her medical checkup. Chest x-ray showed cardiomegaly. Echocardiography and CT angiography showed large Cameral CAF involving the left main and left anterior descending artery draining into the left ventricle. The tricuspid valve was dysplastic, there was secundum ASD, and VSD with a right to left shunt. The patient developed episode of cyanotic spells after crying excessively following a CT angiographic procedure which culminated in respiratory arrest and her demise. She was managed as a case of hypoxic spells in the ICU before her death.

**Conclusion:**

This report unveiled unfamiliar case of Cameral coronary artery fistula with left-to-left shunting, cyanosis, and dysplastic tricuspid valve.

## Background

Congenital coronary fistulas (CAF) are abnormal vascular communications of the coronary arteries with the cardiac chambers or any segment of the systemic or pulmonary circulation. They are uncommon abnormalities, accounting for 0.3% of the CHD [1–3].

Concordant congenital heart defects are not uncommon with CAF. Ventricular and atrial septal defects, Patent ductus arteriosus, Pulmonary atresia with intact ventricular septum, and Tetralogy of fallot are common associations [4]. Low pressure cardiac chambers and less often, the pulmonary arteries, and superior vena cava, are often a site of termination of congenital CAF. Terminating in to the left ventricle is an extremely rare phenomenon [4–8]. According to Sakakibara, CAF is divided by angiography in to two anatomic types, one with a proximal coronary segment dilated with the distal end normal; another the coronary artery dilated over the entire length [9].

CAFs are classified either as Coronary cameral fistula or coronary AV fistula when they drainage in to any cardiac chamber or involving pulmonary artery, coronary sinus, superior and inferior vena cavas, bronchial vessels, and other extracardiac veins respectively [10]. CAFs are usually asymptomatic in the first two decades, especially when they are hemodynamically small. The frequency of both symptoms and complications increases after the first two decades. ‘Stealing’ from the adjacent myocardium causing myocardial ischemia, thrombosis and embolism, cardiac failure, atrial fibrillation, rupture, endocarditis/endarteritis, and arrhythmias are complications commonly encountered. Patients with large left-to-right shunts may develop heart failure, especially during infancy [7].

## Case presentation

A 5 months old female infant presented with fast and labored breathing, grunting, and bluish discoloration of the lips and extremities. The bluish discoloration was noticed by the mother at birth and it worsened with crying. The perinatal history was unremarkable. There was no family history of consanguinity, cardiac illness or sudden cardiac death. Vital signs were normal except she was having tachycardia. Oxygen saturation was 64% (atmospheric air). She had wasting, flaring of the ala-nasi, central cyanosis, and grade I clubbing. There was an inter-costal retraction, but the air entry was good bilaterally. Peripheral pulses were palpable and full in volume. The precordium was quiet with the point of maximal impulse at the 4th intercostal space medial to the midclavicular line. There was a fairly audible holo-systolic murmur at the left lower sternal border radiating to the back. There was no organomegaly. The capillary refill time was less than 2 s.

**Fig. 1:**
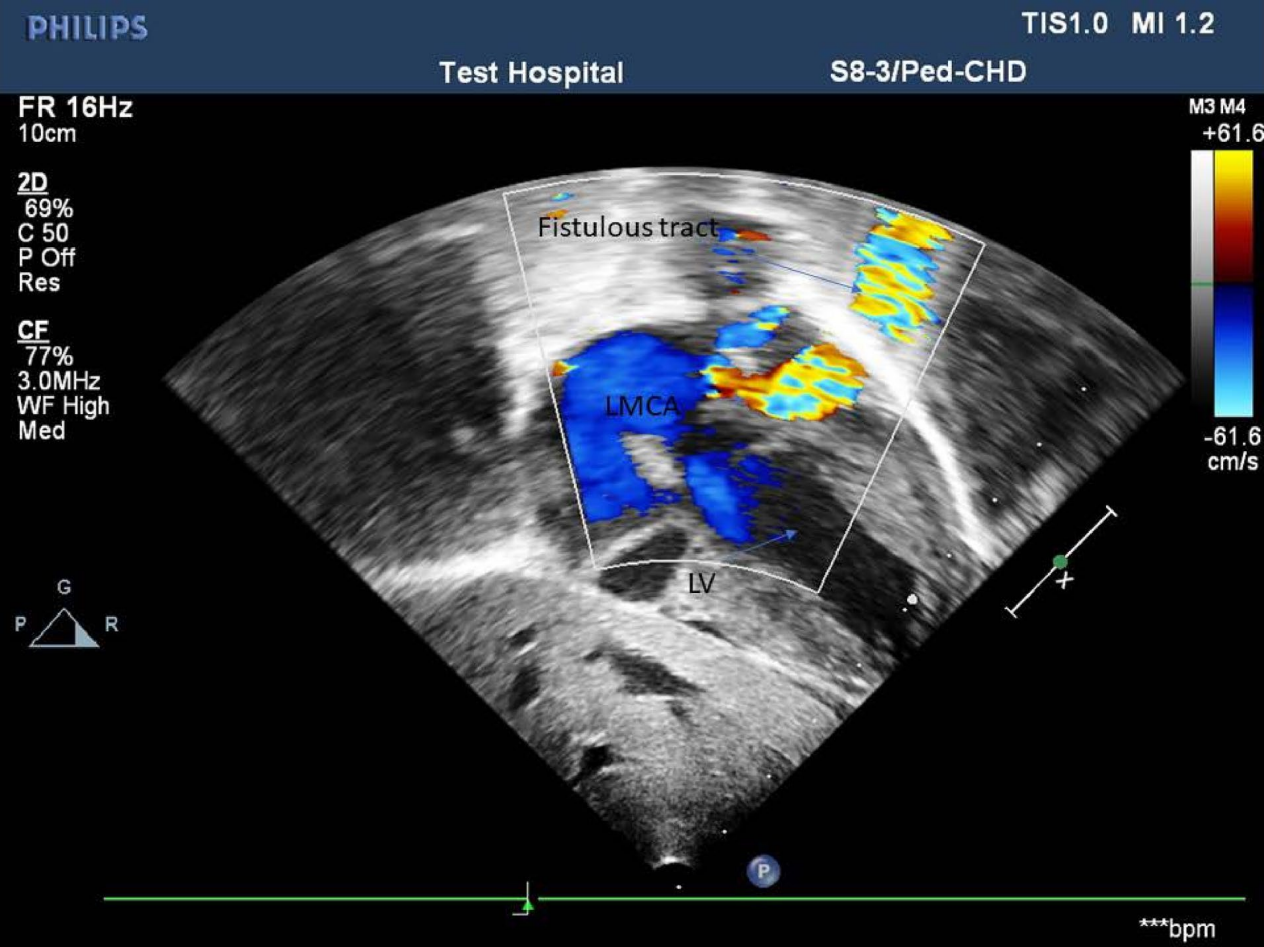
.

**Fig. 2:**
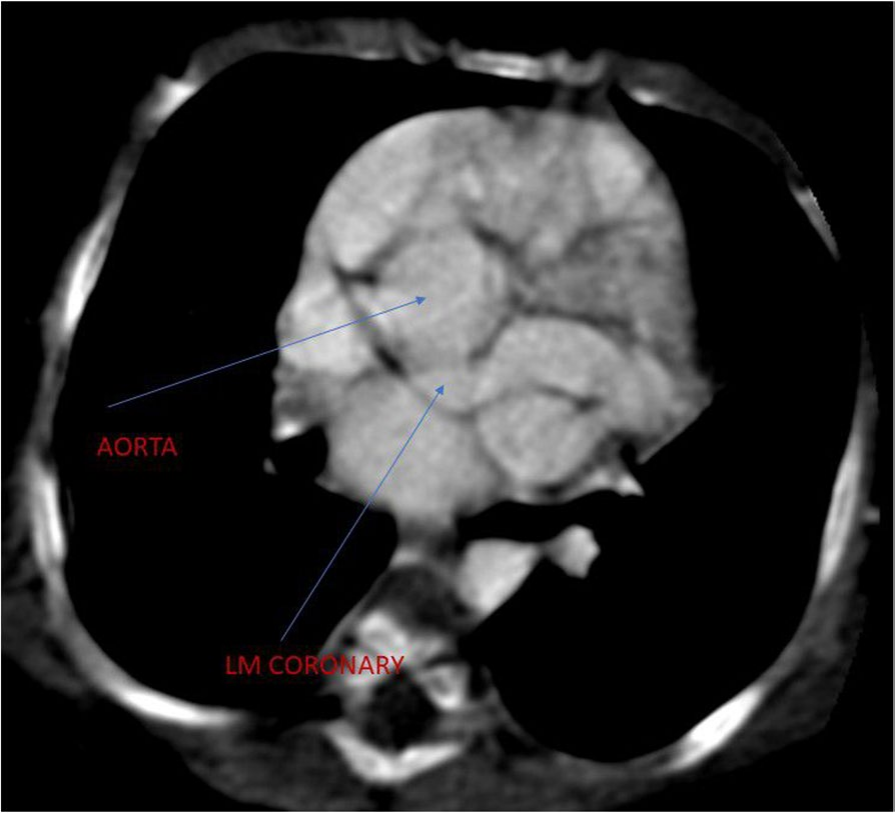
.

**Fig. 3:**
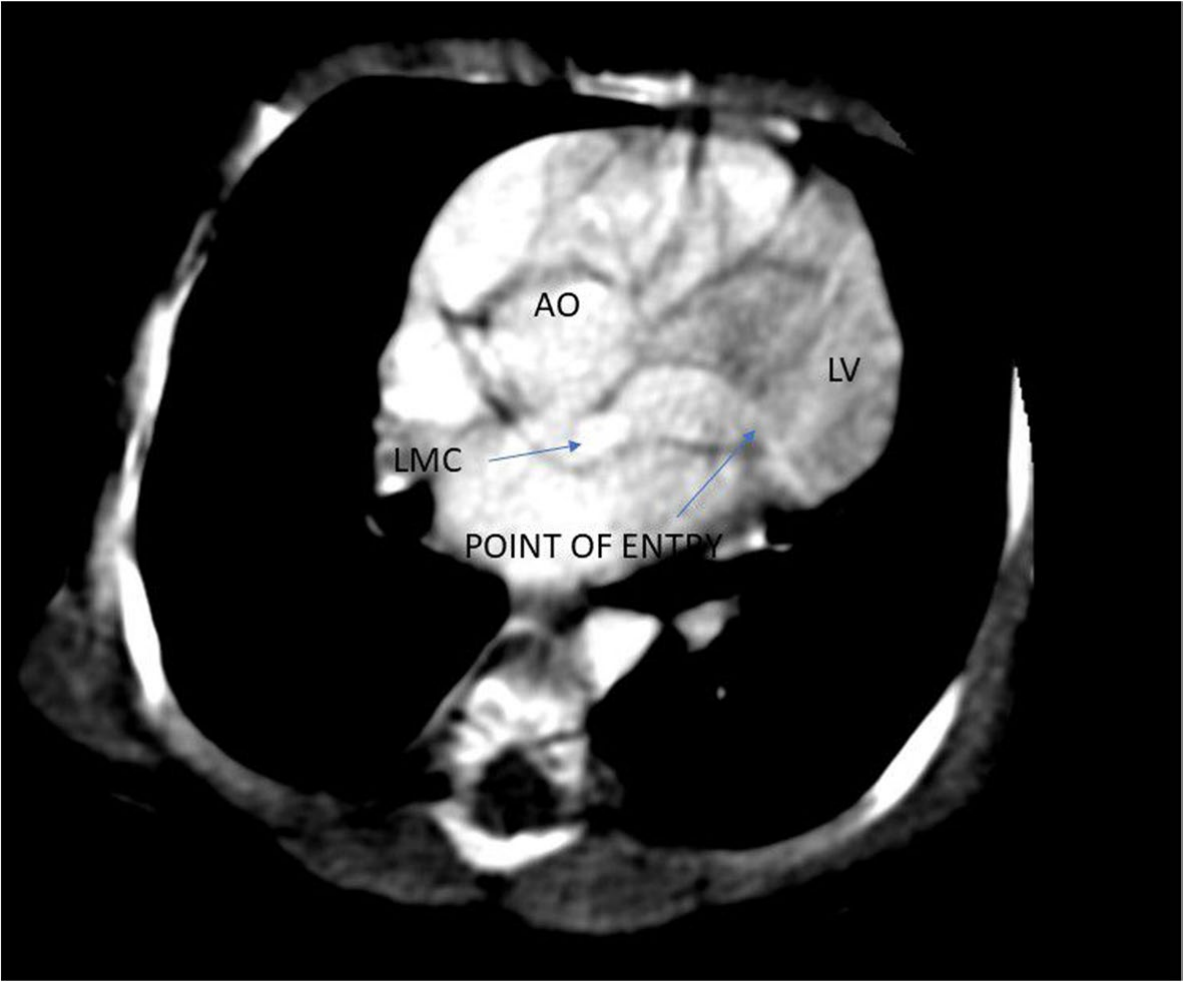
.

**Fig. 4:**
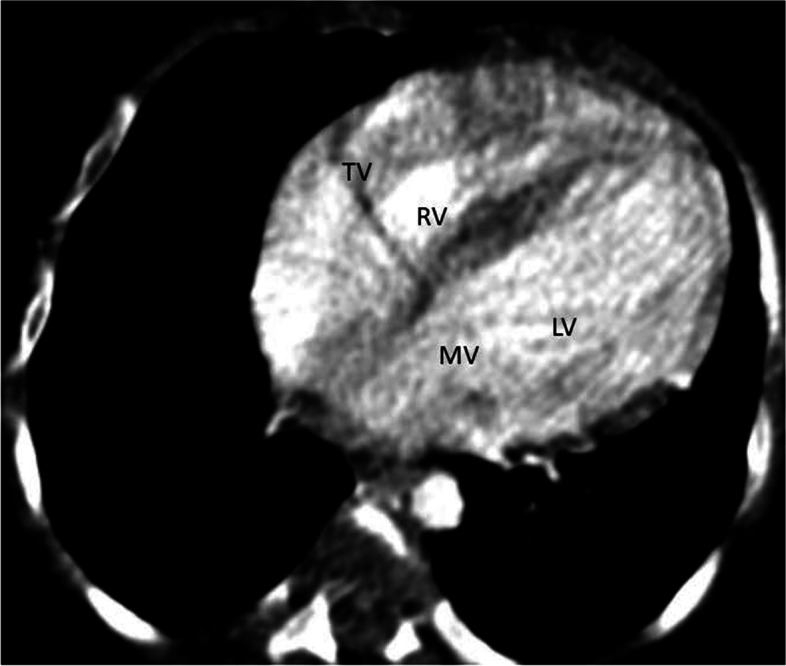
.

Coarse in the hospital were marked with an episode of respiratory arrest, bradycardia and hypoglycemia (admission RBS- 43 mg/dl), for which bag-mask ventilation, chest compression, and bolus IV dextrose (2 ml/kg) was given. She was put on respiratory support. Subsequently, Random blood sugar was 145 mg/dl, WBC—14, 400/mm3, hematocrit- 41.9%, platelet count -97,000/mm3,C- reactive protein of 8 IU/L. Liver function, renal function and serum electrolyte tests were all with in normal limit. Blood culture grew once coagulase-negative staphylococcus aureus. Chest X-ray showed cardiomegaly with a cardiothoracic ratio (CTR) of 66%, reduced vascular markings in both lungs field. Echocardiographic examination showed Cameral coronary artery fistula involving the left main artery draining into the left ventricle. There was also secundum ASD, VSD with a right to left shunt, and tricuspid valve dysplasia (Fig. 1). Chest CT-scan- showed a markedly dilated left aortic sinus, and left main coronary artery involving the left anterior descending branch entering on the lateral wall of the left ventricle. (Figs. 2 and 3). Figure 4 showed atretic tricuspid valve with hypoplastic right ventricle. She had a repeated episode of the cyanotic spells specially following excessive crying. Her oxygen saturation (SaO2) dropped to 40% while she had protracted acidotic breathing for which she was given oxygen by facemask, sodium bicarbonate 1 meq/kg IV before she developed cardio- respiratory arrest and died despite the resuscitative measures.

## Discussion

Our patient had left to left shunt via a tortuous CAF with cyanosis, clubbing of the fingers and frequent hypoxic spells. According to Haller and Little, the clinical triads of a CAF are a cardiac murmur, septal defects with a left-to-right shunt, and a large tortuous coronary artery [9]. Left to left shunt with CAF is rarely reported in children. Hsieh K-S et al. described 10 cases of Coronary artery fistula in neonates, infants and children where, in all the 10 cases the CAF terminated to the right side chambers and arteries (7 cases to right ventricle,2 cases to right atrium and 1 case to the pulmonary artery) and all cases presented with congestive heart failure [11].

Our patient did not show features of heart failure. We have not also found a pediatric case report discussing CAF with a left-to-left shunt having atrio-ventricular valve dysplasia at least to our knowledge. Sigita Glaveckaite et al. reported a 42-year-old women with CAF involving both the Left Main and left circumflex artery communicating with the left ventricle. Their patient had clinical features of left heart failure [12].

In contrast, our patient had a left to left shunt CAF, with frequent hypoxic spells and cardiac arrest. The frequent hypoxic spells probably is due to the right to left shunt at the atrial level and the presence of severe pulmonary hypertension. Severe symptom in children with CAF has been described based on the severity of steal phenomena secondary to a left-to-right shunt between the high (coronary artery) and the low-pressure beds (eg the right cardiac chambers). No similar case has been described in relation to our report at least to our knowledge. Congenital CAF associated with severe right or left outflow tract obstruction has been described [13].

Our patient had atrio-ventricular inflow obstruction, not outflow obstruction and that may be due to the dysplastic tricuspid valve. The cyanosis can be explained by the atretic tricuspid valve and the right to left shunt across the obligatory ASD. Although, death was reported rarely in CAF, our patient succumbed due to frequent hypoxic spells [11]. The CT angiographic procedure may have precipitated the cyanotic spells and caused the respiratory arrest and death. A lesson is learned that subjecting such a precarious patient to an inconvenient procedure like a CT scanning could be catastrophic.

## Conclusion

The report demonstrated a rare form of cameral CAF in an infant who died of it. The uncommon associated cardiac anomaly has emphasized of its importance clinically as well as educationally.

## Data Availability

All available data are within the manuscript.

## Abbreviations

RCA: Right coronary artery
RV: Right ventricle
CAF: Coronary artery fistula
LADA: Left anterior descending artery
CHD: Congenital heart disease
RPDA: Right posterior descending artery
TOF: Tetralogy of Fallot
OM: Obtuse marginal
ASD: Atrial septal defect
D: Diagonal
PDA: Patent ductus arteriosus
VSD: Ventricular septal defect
RA: Right atrium
PAIVS: Pulmonary atresia with intact ventricular septum
CT scan: Computerized tomographic scan
PA: Pulmonary arteries
LV: Left ventricle
